# The vagus nerve-dependent lung-brain axis mediates brain demyelination following acute lung injury

**DOI:** 10.1016/j.bbih.2025.100966

**Published:** 2025-02-11

**Authors:** Dan Xu, Mingming Zhao, Guilin Liu, Tingting Zhu, Yi Cai, Rumi Murayama, Yong Yue, Kenji Hashimoto

**Affiliations:** a Chiba University Center for Forensic Mental Health, Chiba, 260-8670, Japan; b Department of Critical Care Medicine, Union Hospital, Tongji Medical College, Huazhong University of Science and Technology, Wuhan, 430022, PR China; c Institute of Anesthesia and Critical Care Medicine, Union Hospital, Tongji Medical College, Huazhong University of Science and Technology, Wuhan, 430022, PR China; d Department of Anesthesiology, Pain and Perioperative Medicine, The First Affiliated Hospital of Zhengzhou University, Zhengzhou, 450052, PR China; e Department of Pharmacology, Chiba University Graduate School of Medicine, Chiba, 260-8670, Japan; f Department of Anesthesiology, The Affiliated Hospital of Qingdao University, Qingdao, 266100, PR China; g Laboratory of Chemical Pharmacology, Graduate School of Pharmaceutical Sciences, Chiba University, Chiba, 260-8675, Japan

**Keywords:** Cervical vagus nerve, Demyelination, Lung-brain axis, Subdiaphragmatic vagus nerve

## Abstract

Patients with acute lung injury (ALI) often experience psychiatric and neurological symptoms; however, the precise underlying mechanisms remain unclear. Given that white matter loss (demyelination) contributes to these symptoms, we investigated whether lipopolysaccharide (LPS)-induced ALI leads to brain demyelination via a vagus nerve-dependent lung-brain axis. A single intratracheal injection of LPS caused severe lung injury and demyelination in the corpus callosum (CC) of mouse brains. Subdiaphragmatic vagotomy did not affect LPS-induced lung injury or demyelination in the CC. Interestingly, cervical vagotomy significantly attenuated LPS-induced hypo-locomotion, plasma interleukin-6 levels, and demyelination in the CC of ALI mice without influencing lung injury. These findings demonstrate that ALI can induce demyelination in the CC of the mouse brain via a cervical vagus nerve-dependent lung-brain axis, highlighting the critical role of this pathway in the psychiatric and neurological symptoms observed in ALI patients.

## Highlights

• Acute lung injury (ALI) induced demyelination in the mouse brain.
• Subdiaphragmatic vagotomy did not affect brain demyelination in ALI mice.
• Cervical vagotomy blocked brain demyelination in ALI mice.
• ALI caused brain demyelination through a cervical vagus nerve-dependent lung-brain axis.

## 1 Introduction

Acute lung injury (ALI) is characterized by sudden inflammation and damage to the lungs, commonly triggered by factors such as sepsis, pneumonia, trauma, or aspiration. Globally, the incidence of ALI varies widely, estimated at 10 to 86 cases per 100,000 people annually. This variation depends on factors like geographic location, diagnostic criteria, and underlying health conditions (Rubenfeld et al., 2005; Wheeler and Bernard, 2007). ALI is a major contributor to morbidity and mortality in intensive care units, and in severe cases, it frequently progresses to acute respiratory distress syndrome. Patients with ALI often experience lasting psychiatric and neurological complications, including delirium, anxiety, depression, and cognitive impairment. These issues can persist long after the initial lung injury (Hopkins et al., 1999; Huang et al., 2016). These complications are believed to result from systemic inflammation, hypoxia, and prolonged intensive care, all of which affect brain function and mental health. Over time, these effects can considerably reduce quality of life and create challenges in rehabilitation and recovery.

Numerous clinical studies have reported that patients infected with SARS-CoV-2 exhibit white matter loss (demyelination) in their brains, potentially leading to long-term psychiatric and neurological symptoms in survivors (Huang et al., 2022; Lu et al., 2020). This demyelination may be associated with factors such as inflammation, immune dysregulation, and direct viral effects on the central nervous system (CNS) (Lu et al., 2020), contributing to long-COVID symptoms like cognitive impairment and fatigue (Hashimoto, 2023b; Ismail and Salama, 2022). Although ALI is known to produce various psychiatric and neurological symptoms, the exact mechanisms linking ALI to brain demyelination remain unclear.

Given the influence of the lung-brain axis on psychiatric and neurological conditions (Bajinka et al., 2022; Park and Lee, 2024), we investigated whether ALI can induce brain demyelination in mice through this pathway. We further explored the effects of subdiaphragmatic vagotomy (SDV) and cervical vagotomy on ALI-induced brain demyelination, as the vagus nerve plays a central role in the bidirectional communication between the brain and peripheral organs, including the lungs (Ma et al., 2025).

## 2 Methods

### 2.1 Animals

Male adult C57BL/6 mice, aged 8 weeks and weighing 20–25g (Japan SLC, Inc., Hamamatsu, Japan), were used for these experiments. The mice were housed in a controlled temperature environment on a 12-h light/dark cycle (lights on from 07:00 to 19:00) with ad libitum access to food (CE-2; CLEA Japan, Inc., Tokyo, Japan) and water. This study was approved by the Chiba University Institutional Animal Care and Use Committee (Approval No. 6–238).

### 2.2 Compounds and treatments

Lipopolysaccharide (LPS: L-4130, serotype 0111, Sigma-Aldrich, St. Louis, MO, USA) was dissolved in saline, with a dose of 5.0 mg/kg administered, as reported previously (Hashimoto et al., 2022). All other reagents were commercially obtained. Mice were divided into four groups: sham + saline, cervical vagotomy (or SDV) + saline, sham + LPS, and cervical vagotomy (or SDV) + LPS. On day 8 (following recovery from cervical vagotomy) or day 15 (following recovery from SDV), LPS (5.0 mg/kg) or saline (2 ml/kg) was administered intratracheally using Natsume Aerosol Sprayers (KN-34-700, Natsume Seisakusho Co., Ltd. Tokyo, Japan) (Hashimoto et al., 2022).

### 2.3 Subdiaphragmatic vagotomy (SDV)

Bilateral SDV was performed under anesthesia with 5% isoflurane, following the procedure previously described (Zhang et al., 2020). Briefly, a 1 cm transverse incision was made on the right side, 0.5 cm below the xiphisternum, beginning at the linea alba. The liver was carefully retracted using a small cotton pellet moistened with sterile saline, and the costal arch was gently lifted with a vascular clamp to expose the esophagus. Under a surgical microscope (Leica, Heidelberg, Germany), the dorsal and ventral branches of the vagus nerve were exposed along the subdiaphragmatic esophagus. Successful SDV was confirmed by observing an increased stomach size 14 days post-operation. For the sham surgery, the vagus nerve was exposed but left intact. In all mice undergoing SDV, extra care was taken to avoid injury to the subdiaphragmatic esophagus. Mice were allowed to recover for 14 days after the bilateral SDV procedure.

### 2.4 Cervical vagotomy

Mice were anesthetized with 5% isoflurane and secured under a stereoscope (Leica, Heidelberg, Germany) using medical tape. The throat was sprayed with 70% ethanol to wet the fur. The skin was lifted, and a 1 cm vertical incision was made on the throat. The right vagal nerve was then carefully isolated and separated from the carotid artery using small, curved forceps. Unilateral cervical vagotomy was performed by lifting and cutting the vagal nerve with straight scissors, as previously reported (Su et al., 2024). For sham operations, the vagal nerve was lifted and then released intact. After the cervical vagotomy, the wounds were sutured and disinfected with povidone-iodine. Mice were allowed a recovery period of seven days before the corresponding experiments were conducted.

### 2.5 Locomotion test

The locomotion test was conducted 24 h after the LPS (5 mg/kg) injection, as previous reported (Xu et al., 2024). Each mouse's locomotor activity was measured using an automated animal movement analysis system (SCANET MV-40; MELQUEST Co., Ltd., Toyama, Japan). Cumulative ambulatory activity was recorded over a 30-min period after placing the mice in experimental cages (560 × 560 × 330 mm). The cages were cleaned between each testing session.

### 2.6 Collection of samples

Under anesthesia with 5% isoflurane, blood was collected via cardiac puncture and placed into tubes containing ethylenediaminetetraacetic acid (EDTA), as described previously (Xu et al., 2024; Zhao et al., 2024). The blood samples were immediately centrifuged at 3000×*g* for 10 min at 4 °C to obtain plasma, which was then stored at −80 °C until further analysis. Next, the inferior lobe of the right lung was collected from each mouse, followed by transcardial perfusion with isotonic saline and ice-cold 4% paraformaldehyde in 0.1 mM phosphate buffer (30 ml per mouse, pH 7.4). The brain was subsequently collected, post-fixed overnight at 4 °C, and sectioned into 30 μm slices using a vibratome (VT1000S, Leica Microsystems AG, Wetzlar, Germany). Slices including corpus callosum (CC) were selected from bregma 1.10 to −0.58 in the mouse brain for immunofluorescence analysis.

### 2.7 Histopathology and immunofluorescence

The inferior lobe of the right lung was collected and fixed in 10% formalin (FUJIFILM Wako Pure Chemical Corporation, Tokyo, Japan). Hematoxylin and eosin (HE) staining was performed by Biopathology Institute Co., Ltd. (Kunisaki, Oita, Japan). Lung samples were embedded in paraffin, sectioned at 5 μm, and stained with HE. Representative images from the four groups were captured using a Keyence BZ-9000 Generation II microscope (Osaka, Japan). Morphological changes were scored as nil (0), mild (1), moderate (2), or severe (3) based on five pathological features: (i) presence of exudates, (ii) hyperemia/congestion, (iii) neutrophil infiltration, (iv) intra-alveolar hemorrhage/debris, and (v) cellular hyperplasia, as described previously (Hashimoto et al., 2022). Scores for each mouse in each group were summed and averaged.

Black-Gold II staining (Black-Gold II RTD Myelin Stain Reagent, catalog number: TR-100-BG, Biosensis, Australia) was performed according to the manufacturer's instructions (Xu et al., 2024; Zhao et al., 2024). Immunofluorescence was performed as previously described (Xu et al., 2024; Zhao et al., 2024). Briefly, brain slices were washed three times with 0.1 mM phosphate buffer for 5 min each, then blocked with 3% BSA and 0.3% Triton X-100 for 2 h. Slices were incubated overnight at 4 °C with the primary antibody [mouse, anti-MBP (myelin basic protein), catalog No. sc-271,524, Santa Cruz Biotechnology, Inc., CA, USA, 1:100]. The following day, slices were incubated with the secondary antibody (Alexa Fluor 546 goat anti-mouse IgG1, 1:1000) for 2 h at room temperature. Afterward, the slices were washed three times in 0.1 mM phosphate buffer containing 0.1% Tween-20 for 5 min each, mounted with a DAPI-containing medium (4′,6′-diamidino-2-phenylindole, catalog No. H1200, Vector Laboratories, Inc., USA), and analyzed using a Keyence BZ-900 microscope (Tokyo, Japan) with Image J software.

### 2.8 Statistical analysis

Data are presented as the mean ± standard error of the mean (S.E.M.). Statistical analyses were performed using PASW Statistics 20 (formerly SPSS Statistics; SPSS) or R software. For comparisons between two groups, either a *t*-test or a non-parametric test was used, depending on data distribution. Comparisons among four groups were conducted using two-way analysis of variance (ANOVA) or a two-way non-parametric test. P-values less than 0.05 were considered statistically significant.

## 3 Results

### 3.1 Effects of ALI on the demyelination in the corpus callosum (CC)

**Fig. 1:**
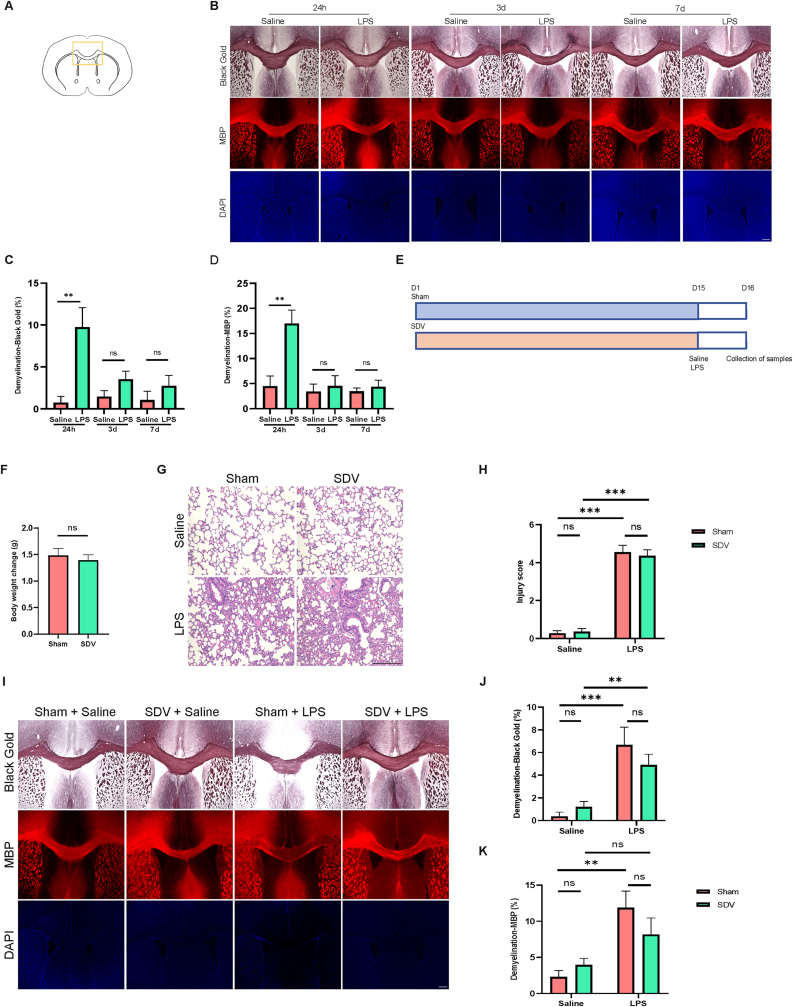
Effects of ALI on demyelination in the CC and the effects of SDV on the demyelination and lung injury following intratracheal administration of LPS (A): Black-Gold II and MBP staining images were taken from the area outlined by the yellow box. (B): Representative images of Black-Gold II staining, MBP, and DAPI in the corpus callosum (CC) of brains from the saline and LPS groups at 24 h, 3 days, and 7 days. (C): Quantitative analysis of the demyelinated area in the CC using Black-Gold II staining at 24 h, 3 days, and 7 days (24 h: Mann-Whitney test, U = 4, P = 0.0087; 3 d: Unpaired *t*-test, t(8) = 1.707, P = 0.1261; 7 d: Mann-Whitney test, U = 1, P = 0.0714). (D): Quantitative analysis of the demyelinated area in the CC using MBP staining (24 h: unpaired *t*-test, t(9) = 3.567, P = 0.0061; 3 d: Unpaired *t*-test, t(8) = 0.4230, P = 0.6834; 7 d: unpaired *t*-test, t(8) = 0.6549, P = 0.5309). (E): Treatment schedule: Adult mice were subjected to subdiaphragmatic vagotomy (SDV) and allowed to recover for 14 days. On day 15, LPS (5.0 mg/kg) or saline (2 ml/kg) was administered intratracheally. Lung and brain samples were collected on day 16. (F): Body weight change (unpaired *t*-test, t(42) = 0.5538, P = 0.5826). (G, H): HE staining of the lungs ( × 200) and histological injury score of the lungs across the four groups, 24 h post-LPS administration (two-way non-parametric test, SDV: H = 0, P = 1; LPS: H = 34.022, P < 0.001; interaction: H = 0.07, P = 0.7909). Scale bar = 200 μm. (I): Representative images of Black-Gold II staining, MBP, and DAPI in the CC from the four experimental groups. Scale bar = 300 μm. (J): Quantitative analysis of the demyelinated area in the CC using Black-Gold II staining (two-way non-parametric test, SDV: H = 0.5261, P = 0.4682; LPS: H = 20.4422, P < 0.001; interaction: H = 0.8441, P = 0.3582). (K): Quantitative analysis of the demyelinated area in the CC using MBP staining (two-way non-parametric test, SDV: H = 0.0050, P = 0.9438; LPS: H = 10.8149, P = 0.001; interaction: H = 3.0216, P = 0.0821). Data are presented as mean ± SEM (A–D: saline group, n = 5 at 24 h, n = 5 at 3 and 7 days; LPS group, n = 6 at 24 h, n = 5 at 3 and 7 days; E–K: sham + saline, SDV + saline, sham + LPS, and SDV + LPS groups, n = 11). ∗∗P < 0.01, ∗∗∗P < 0.001, N.S.: not significant.

Mice were given an intratracheal injection of either LPS (5.0 mg/kg) or saline (2 ml/kg), and demyelination in the corpus callosum (CC) was assessed at 1, 3, and 7 days post-administration. Demyelination areas in the brain were evaluated using Black-Gold II staining and MBP immunostaining (Fig. 1A). Compared to the saline group, the LPS-treated group exhibited significantly greater demyelination on day 1 (Fig. 1B, C, D). No significant differences in demyelination were found between the two groups on days 3 and 7 (Fig. 1B, C, D). These results indicate that LPS-induced ALI led to demyelination in the CC one day after a single intratracheal injection.

### 3.2 Effects of SDV on demyelination in the CC and lung injury after LPS administration

To examine the effects of SDV on LPS-induced demyelination in the CC and lung injury, LPS or saline was administered intratracheally on day 15 post-SDV or sham surgery (Fig. 1E). SDV had no effect on the body weight of mice before LPS injection (Fig. 1F). Lung tissues in the LPS-treated groups exhibited severe pathological changes compared to those in the saline-treated group (Fig. 1G), with a significantly higher lung injury score in the LPS-treated group than in the saline-treated group (Fig. 1H). However, there was no reduction in lung injury score in the SDV + LPS group compared to the sham + LPS group (Fig. 1H).

The LPS-treated group showed significant demyelination in the CC compared to the saline-treated group, and the SDV + LPS group did not show a reduction in demyelination area compared to the sham + LPS group, although SDV slightly decreased demyelination in the CC of LPS-treated mice (Fig. 1I, J, K).

Collectively, these findings indicate that SDV does not impact demyelination in the CC or lung injury following LPS administration, suggesting that the subdiaphragmatic vagus nerve does not play a crucial role in LPS-induced demyelination in the CC.

### 3.3 Effects of cervical vagotomy on demyelination in the CC, lung injury, locomotion, and plasma IL-6 levels after LPS administration

**Fig. 2:**
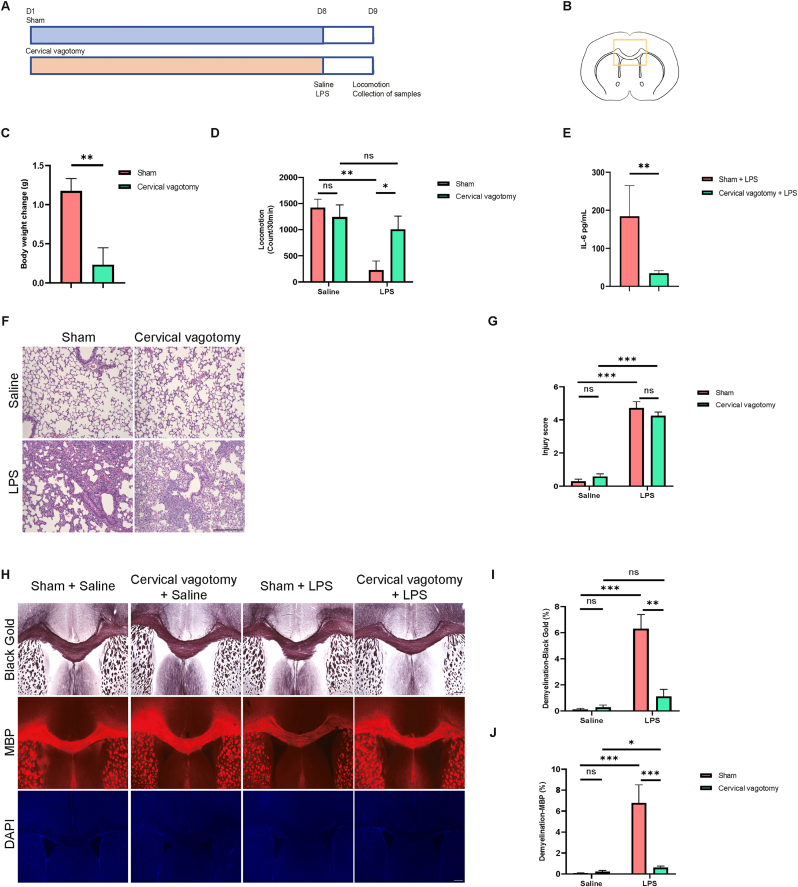
Effects of cervical vagotomy on demyelination in the CC, lung injury, locomotion, and plasma IL-6 levels following intratracheal administration of LPS (A): Treatment schedule: Adult mice underwent cervical vagotomy or sham surgery and were allowed to recover for 7 days. On day 8, LPS (5.0 mg/kg) or saline (2 ml/kg) was administered intratracheally. Plasma, lung, and brain samples were collected on day 9 after the locomotion test. (B): Black-Gold II and MBP staining images were taken from the area outlined by the yellow box. (C): Body weight change before injection of saline or LPS (unpaired *t*-test, t(32) = 3.420, P = 0.0017). (D): Locomotion test results (two-way non-parametric test, cervical vagotomy: H = 0.2239, P = 0.636; LPS: H = 10.0061, P = 0.0015; interaction: H = 4.1224, P = 0.0423). (E): Plasma IL-6 levels (Mann-Whitney test, U = 3, P = 0.0047). (F, G): HE staining of the lung ( × 200) and lung histological injury scores for the four groups, 24 h post-LPS administration (two-way non-parametric test, cervical vagotomy: H = 0.0018, P = 0.9665; LPS: H = 21.8849, P < 0.001; interaction: H = 1.1028, P = 0.2937). Scale bar = 200 μm. (H): Representative images of Black-Gold II staining, MBP, and DAPI in the CC across the four groups. Scale bar = 300 μm. (I): Quantitative analysis of demyelination area in the CC using Black-Gold II staining (two-way non-parametric test, cervical vagotomy: H = 1.4465, P = 0.2291; LPS: H = 14.5047, P < 0.001; interaction: H = 3.7038, P = 0.0543). (J): Quantitative analysis of demyelination area in the CC using MBP staining (two-way non-parametric test, cervical vagotomy: H = 0.8718, P = 0.3505; LPS: H = 16.5907, P < 0.001; interaction: H = 3.5874, P = 0.0582). Data are presented as mean ± SEM. Body weight: sham (n = 16), and cervical vagotomy (n = 18). Locomotion test: sham + saline (n = 9), cervical vagotomy + saline (n = 9), sham + LPS (n = 7), and cervical vagotomy + LPS (n = 9). Plasma IL-6 levels: sham + LPS (n = 6), and cervical vagotomy + LPS (n = 8). Histological injury score and demyelination area using Black-Gold II and MBP staining: sham + saline (n = 8), cervical vagotomy + saline (n = 8), sham + LPS (n = 6), and cervical vagotomy + LPS (n = 8). ∗P < 0.05, ∗∗P < 0.01, ∗∗∗P < 0.001, N.S.: not significant.

To investigate the effects of cervical vagotomy on LPS-induced demyelination in the CC and lung injury, LPS or saline was administered intratracheally on day 8 post-vagotomy or sham surgery (Fig. 2A and B). Cervical vagotomy significantly attenuated the postoperative body weight increase (Fig. 2C). In the locomotion test, the sham + LPS group showed a significant decrease in motor function compared to the sham + saline group, whereas the cervical vagotomy + LPS group demonstrated a significant improvement in motor function compared to the sham + LPS group (Fig. 2D). Additionally, plasma IL-6 levels were significantly lower in the cervical vagotomy + LPS group than in the sham + LPS group (Fig. 2E).

Lung tissues in the LPS-treated groups showed severe pathological changes compared to the saline-treated group (Fig. 2F), with a significantly higher lung injury score in the LPS-treated group than in the saline-treated group (Fig. 2G). However, there was no difference in lung injury scores between the cervical vagotomy + LPS and sham + LPS groups (Fig. 2G). The sham + LPS group displayed significant demyelination in the CC compared to the sham + saline group (Fig. 2H, I, J). Interestingly, the cervical vagotomy + LPS group exhibited a reduction in the demyelination area compared to the sham + LPS group (Fig. 2H, I, J).

Overall, these findings suggest that cervical vagotomy alleviated LPS-induced increases in plasma IL-6 levels, reductions in motor function, and demyelination in the CC, but had no effect on LPS-induced lung injury.

## 4 Discussion

In this study, we found several novel results. First, a single intratracheal injection of LPS caused demyelination in the CC of the mouse brain along with severe lung injury. Second, SDV did not alter LPS-induced demyelination in the CC or lung injury, suggesting that the subdiaphragmatic vagus nerve does not play a critical role in ALI-induced demyelination via the lung-brain axis. Third, cervical vagotomy significantly reduced hypo-locomotor activity, plasma IL-6 levels, and demyelination in the CC of ALI mice compared to sham-operated controls. These results indicate that LPS-induced ALI can lead to demyelination in the mouse brain through a cervical vagus nerve-dependent lung-brain axis. The contrasting effects observed between SDV and cervical vagotomy suggest that, in the intratracheal LPS model, the cervical vagus nerve is more critical than its subdiaphragmatic branches in mediating the systemic inflammatory response that contributes to brain demyelination.

In this study, we observed demyelination in the CC of ALI mice just 24 h after a single LPS injection. However, no further changes in demyelination were detected at 3 and 7 days post-injection. These findings suggest that remyelination initiates rapidly, despite the typical process involving myelin debris clearance, oligodendrocyte precursor cell (OPC) recruitment, and subsequent myelin sheath formation. Overall, our data indicate an early activation of repair mechanisms by day 3, with full remyelination evident by day 7, consistent with the expected biological sequence.

In our previous study, we found that SDV significantly reduced depression-like behavior, plasma IL-6 levels, and altered gut microbiota composition following a single intraperitoneal injection of low-dose LPS (0.5 mg/kg) (Zhang et al., 2020), underscoring the critical role of the subdiaphragmatic vagus nerve in systemic inflammation. In the current study, we observed that cervical vagotomy attenuated postoperative weight gain, whereas SDV had no significant effect. This difference may be attributed to SDV-induced stomach dilation, which can increase the volume of gastrointestinal contents and thus body weight, while cervical vagotomy might impair swallowing, leading to reduced food intake and less weight gain. Additionally, cervical vagotomy significantly reduced LPS (5 mg/kg)-induced hypo-locomotion and plasma IL-6 levels, whereas neither cervical vagotomy nor SDV affected LPS-induced lung injury. Collectively, these findings suggest that the cervical vagus nerve, rather than the subdiaphragmatic vagus nerve, plays a crucial role in mediating systemic inflammation in mice with ALI.

In this study, we observed that cervical vagotomy significantly reduced plasma IL-6 levels and demyelination in the CC of ALI mice. Previously, we demonstrated that intravenous injection of an anti-mouse IL-6 receptor antibody (MR16-1) produced rapid, long-lasting antidepressant effects in mice susceptible to chronic social defeat stress (CSDS), effects attributed to its anti-inflammatory properties (Zhang et al., 2017). In contrast, intracerebroventricular injection of MR16-1 did not yield antidepressant benefits in CSDS-susceptible mice, suggesting that peripheral IL-6 plays a key role in mediating depression-like behaviors in the CSDS model. However, it remains unclear from our current data whether the reduction of blood IL-6 via cervical vagotomy directly contributes to the observed decrease in demyelination in ALI mice. It would be valuable to determine if treatment with a neutralizing antibody against IL-6 can similarly affect demyelination in the CC.

Beyond the well-known role of gut microbiota, lung microbiota may also significantly influence health and disease (Hashimoto, 2023a). One study demonstrated that disruption of the lung microbiota following repeated intratracheal administration of the antibiotic neomycin affected rats’ susceptibility to CNS autoimmune diseases in an experimental autoimmune encephalomyelitis model (Hosang et al., 2022). Additionally, this study showed that a well-balanced lung microbiota can modulate microglial activation in the CNS by increasing the colonization of LPS-producing bacterial taxa. However, the role of the vagus nerve was not explored in this context. Further research is needed to investigate how the vagus nerve might mediate the relationship between lung microbiota and brain autoimmunity.

The transient demyelination seen in our mouse model may differ in duration and severity from that in human ALI patients. Although some neurological deficits in ALI patients are temporary, reports indicate persistent neurocognitive and neuropsychiatric impairments (Sasannejad et al., 2019). The progression of neurological symptoms following ALI likely depends on factors such as lung injury severity, comorbid conditions, and repeated inflammatory episodes. Even though the changes in our mouse model are transient, they underscore the importance of the lung-brain axis in triggering early brain alterations after ALI. This finding enhances our understanding of how acute systemic insults can initiate neurological dysfunction—even if only temporarily in experimental settings—and suggests that more severe or prolonged injuries in humans might cause lasting damage. However, these results may not fully translate to clinical ALI cases, and further prospective clinical studies are needed to determine whether similar changes occur in ALI patients.

Although the exact signaling pathways remain to be fully elucidated, our working model suggests that vagal activation may trigger the release of pro-inflammatory cytokines, such as IL-6, or other mediators within the CNS that negatively impact oligodendrocytes and myelin integrity. It is also unclear whether the demyelination observed in our model is adaptive or maladaptive. One possibility is that transient demyelination serves as an acute stress response, allowing the brain to rapidly activate repair and protective mechanisms in the face of systemic inflammation. Conversely, if the inflammatory insult is too severe or prolonged, these changes could become maladaptive, resulting in persistent neurocognitive deficits.

Demyelination, the loss of the myelin sheath surrounding nerve fibers, disrupts efficient neural communication, which is essential for normal brain function. This disruption can result in cognitive impairments, mood disorders, and other neurological symptoms commonly seen in psychiatric conditions (Nave and Ehrenreich, 2014). In this study, we found that cervical vagotomy significantly reduced LPS-induced demyelination in the CC of the mouse brain via the lung-brain axis. These results suggest that the cervical vagus nerve plays a role in the psychiatric and neurological symptoms observed in ALI patients. Moreover, given the anti-inflammatory effects of non-invasive transcutaneous auricular vagus nerve stimulation (Ma et al., 2025), this technique may hold therapeutic potential for alleviating these symptoms in ALI patients.

This study has several limitations. First, we focused on demyelination in the CC due to its high myelin concentration, but other regions—such as the hippocampus—are critical for neurological and psychiatric functions. Future studies should examine the involvement of the cervical vagus nerve-dependent lung-brain axis in these areas affected by ALI. Second, although neurocognitive impairments are reported in ALI patients, we did not assess cognitive deficits in our animal model through behavioral tests. Further research should explore the role of the vagus nerve in these cognitive impairments. Third, the molecular and cellular mechanisms linking ALI-induced dysbiosis of the lung microbiota to brain demyelination remain unclear. Finally, our model is designed not to imply that ALI causes a full demyelinating syndrome, but rather to study early neuroinflammatory responses to lung injury. Our findings suggest that ALI-induced systemic inflammation may lead to subtle white matter changes—such as the transient demyelination observed in the CC. Additional studies are necessary to elucidate the complex pathways connecting ALI with psychiatric and neurological symptoms.

This study demonstrates that ALI induced by intratracheal LPS leads to rapid demyelination in the CC, a process mediated by the cervical vagus nerve. These findings suggest that targeting vagal pathways may offer a promising strategy to alleviate neuroinflammation and subsequent neurological impairments associated with lung injury.

## CRediT authorship contribution statement

**Dan Xu:** Conceptualization, Data curation, Formal analysis, Investigation, Writing – original draft, Writing – review & editing. **Mingming Zhao:** Investigation, Writing – review & editing. **Guilin Liu:** Investigation, Writing – review & editing. **Tingting Zhu:** Investigation, Writing – review & editing. **Yi Cai:** Investigation, Writing – review & editing. **Rumi Murayama:** Investigation, Writing – review & editing. **Yong Yue:** Investigation, Writing – review & editing. **Kenji Hashimoto:** Conceptualization, Funding acquisition, Supervision, Writing – original draft, Writing – review & editing.

## Declaration of competing interest

The authors declare that they have no known competing financial interests or personal relationships that could have appeared to influence the work reported in this paper.

## Data Availability

Data will be made available on request.
